# Metagenomic Characterization and Comparative Analysis of Removable Denture-Wearing and Non-Denture-Wearing Individuals in Healthy and Diseased Periodontal Conditions

**DOI:** 10.3390/microorganisms12061197

**Published:** 2024-06-13

**Authors:** Ho-Hin Wong, Chun-Ho Hung, Jason Yip, Tong-Wah Lim

**Affiliations:** Faculty of Dentistry, The University of Hong Kong, Hong Kong SAR, China; jackyhhw@gmail.com (H.-H.W.); leohung@connect.hku.hk (C.-H.H.); u3571854@connect.hku.hk (J.Y.)

**Keywords:** removable denture, metagenomics, pathogenic bacteria, microbial index

## Abstract

Removable denture wearers are at an increased risk of developing periodontal diseases due to biofilm deposition and microbial colonization on the denture surface. This study aimed to characterize and compare the metagenomic composition of saliva in denture wearers with different periodontal statuses. Twenty-four community-dwelling elders were recruited and grouped into denture wearers with active periodontitis (APD), non-denture wearers with active periodontitis (APXD), denture wearers with stable periodontal health conditions (SPCD), and non-denture wearers with stable periodontal health conditions (SPCXD). Saliva samples were collected and underwent Type IIB restriction-site-associated DNA for microbiome (2bRAD-M) metagenomic sequencing to characterize the species-resolved microbial composition. Alpha diversity analysis based on the Shannon index revealed no significant difference between groups. Beta diversity analysis using the Jaccard distance matrix was nearly significantly different between denture-wearing and non-denture-wearing groups (*p* = 0.075). Some respiratory pathogens, including *Streptococcus agalactiae* and *Streptococcus pneumoniae*, were detected as the top 30 species in saliva samples. Additionally, LEfSe analysis revealed a substantial presence of pathogenic bacteria in denture groups. In the cohort of saliva samples collected from community-dwelling elders, a remarkable abundance of certain opportunistic pathogens was detected in the microbial community.

## 1. Introduction

The ecological plaque hypothesis states that plaque-mediated diseases result from imbalances in the resident microbiota within a particular niche [1]. Non-shedding surfaces in the oral cavity, including teeth and removable dentures, promote bacteria adherence, leading to extensive biofilm deposition. The oral health of patients after the placement of removable partial dentures has been associated with a higher prevalence of plaque accumulation, bleeding on probing, and clinical attachment loss due to inadequate oral hygiene. This can lead to caries, periodontal diseases, halitosis, and denture stomatitis [2,3,4,5]. In addition, pathogenic microorganisms from denture biofilm may be associated with significant systemic health diseases, including bacterial endocarditis, gastrointestinal infections, and respiratory infections [6].

The oral microbiome of healthy individuals showed a high abundance of *Veillonella*, *Neisseria*, *Streptococcus*, and *Prevotella* genera. These are relatively similar among healthy individuals at the genus level [7]. Subgingival microbiomes of patients with diabetes and non-diabetics, presenting with different periodontal conditions, were assessed. It was found that patients with periodontitis exhibited a significantly higher abundance of *Anaerolineaceae bacterium oral taxon 439* compared to the group without periodontitis, using whole-metagenomic shotgun sequencing [8]. In the case of periodontitis, the dynamic interactions between the periodontopathogenic bacteria and host factors, including host immune–inflammatory response, have led to a synergic action of dysbiotic microbial communities, subsequently developing in periodontal diseases [9]. In addition, periodontopathogenic bacteria, including *Porphyromonas gingivalis* and *Filifactor alocis*, were detected at a significantly higher relative abundance in patients with periodontitis. Notably, the oral microbiome has been suggested to play a significant role in the etiology of various systemic diseases, including endocrine disease (Type II diabetes mellitus), respiratory infections, gastrointestinal diseases, and cancers [10,11,12,13].

In the past, the role of *Candida* spp. in the pathogenesis of denture stomatitis was extensively investigated [14]. Therefore, microbiological studies on denture biofilm have focused on fungal composition using culture-based methods. However, in recent times, there has been a limited number of microbiome studies conducted on removable denture wearers [14]. Oral microbiomes among palatal mucosa, removable dentures, and dental plaque were found to form distinct clusters and exhibit compositional differences [15]. The microbial diversity between healthy individuals and those with denture stomatitis showed no statistically significant difference when analyzed using 16S rRNA sequencing [16]. Denture cleanliness was found to affect the microbial community composition, particularly the increased microbial index of pathogenic bacteria (MIP) [17]. Notably, several studies have also demonstrated a high prevalence of respiratory pathogens residing on removable denture surfaces [18,19,20,21].

Metagenomic sequencing techniques have been employed to uncover hidden microbial communities within the oral cavity that are non-cultivable, shedding light on factors that change the oral microbiome and its relationship with oral and systemic health [22,23]. However, most oral microbiome-related studies have focused on dental plaque and used 16S rRNA sequencing [14]. In principle, the 16S rRNA gene sequencing method relies on nucleotide diversity within a single functional gene for microbial identification, which does not resolve species-level differences between microbes, probably leading to low sensitivity [22]. Today, Type IIB restriction-site-associated DNA sequencing for microbiome (2bRAD-M) has gained popularity in profiling bacterial and fungal communities in a cultivation-independent manner, providing quantitative and qualitative data by demonstrating and resolving species-level taxonomy [22]. This method relies on DNA digestion using Type IIB restriction enzyme (BcgI) and the unique 2bRAD tags mapped with the Genome Taxonomy Database (GTDB R202), which established a taxonomy system based on genome-wide distance [24]. In addition, microbiome-based indices were developed using this sequencing technique, based on 300 published categories of opportunistic pathogenic bacteria by the Chinese Center for Disease Control and Prevention, enabling risk assessment for skin, respiratory, gastrointestinal, and oral health [17,18,23,25,26,27]. Particularly, these indices were utilized to profile overall pathogenic bacteria and respiratory pathogens in removable denture biofilm and their relationships with denture cleanliness [17,18].

Currently, the complete microbiological and pathogenic bacterial profiles of denture-wearing and non-denture-wearing individuals in healthy and diseased periodontal conditions are unknown. To understand the polymicrobial nature of periodontitis and the potential for removable dentures to act as reservoirs for opportunistic pathogens, the present study aimed to characterize the oral microbiome, comparing the taxonomic profiles and MIP of denture and non-denture wearers with different periodontal statuses using a high-resolution metagenomic sequencing method (2bRAD-M).

## 2. Materials and Methods

This pilot cross-sectional study was conducted at the Clinical Research Centre, Faculty of Dentistry at The University of Hong Kong, from May 2023 to July 2023. The sample size was determined using the rule of thumb for pilot studies, which recommends 12 participants per group [28]. Following ethical clearance (IRB Reference Number: UW22-256), 24 participants were enrolled after providing written informed consent. 

Participants who complied with the inclusion and exclusion criteria (Table 1) were recruited using the convenience sampling method. All participants aged 60 and above were allocated into two main groups (denture-wearing and non-denture-wearing). These groups were further subdivided into 4 groups: denture wearers with active periodontitis (APD), non-denture wearers with active periodontitis (APXD), denture wearers with a stable periodontal health condition (SPCD), and non-denture wearers with a stable periodontal health condition (SPCXD). Furthermore, all participants’ systemic diseases were effectively managed with medication, except for those mentioned in the exclusion criteria.

Saliva samples were collected from participants according to the protocols recommended by Lim et al. [29]. Participants were asked to abstain from eating and drinking for an hour prior to saliva sample-taking. They were asked to rinse with 10 mL of 0.9% (*w*/*v*) saline solution for 1 min. Afterward, they were instructed to expel the solution into a 50 mL sterile Falcon tube. The collected saliva samples were promptly transferred to the Central Research Laboratories for processing. Samples were centrifuged at 9880 rpm (14,000× *g*) for 10 min, and the remaining plaque pellets were resuspended in 180 μL of 20 mM Tris-Hcl; 2 mM EDTA; 1.2% Triton with 20 μL of 20 mg/mL lysozyme and incubated overnight at 37 °C. Then, 20 μL of proteinase K extraction buffer was added and mixed by vortexing. Saliva samples were incubated at 56 °C for 2 h, followed by 95 °C for 15 min. Ethanol (200 μL) was then added to the sample and mixed by pulse-vortexing for 15 s. Then, the DNA was extracted using the QIAmp Mini DNA Extraction Kit (Qiagen GmbH, Hilden, Germany). DNA concentration was quantified by the Qubit 2.0 Fluorometer (Life Technologies, Carlsbad, CA, USA), and all samples were stored at −70 °C.

All DNA samples were transferred to Qingdao OE Biotech Co., Ltd. (Qingdao, China) under cool conditions. Library construction and metagenomic sequencing were performed by using the principle of 2bRAD-M. The DNA, once extracted, was subjected to digestion with 4 U Type IIB restriction enzyme (BcgI) at a stable temperature of 37 °C for three hours. A reaction mixture was incorporated with 10 μL of the digested product, T4 DNA ligase buffer, 1 mM ATP (NEB), 0.2 μM of adaptors (Ada1 and Ada2), and 800 U of T4 DNA ligase (NEB). This mixture underwent a ligation reaction for an extended period of 16 h followed by an enzyme heat inactivation process at 65 °C lasting 20 min. The subsequent phase involved amplification for DNA sequencing via polymerase chain reaction (PCR) in a designated reaction volume of 40 μL. The PCR mixture composition included 7 μL of ligated DNA, Phusion HF buffer, primers for Illumina at a concentration of 0.1 μM, dNTP at a concentration of 0.3 mM, and Phusion high-fidelity DNA polymerase (NEB) at a concentration of 0.4 U. The PCR reactions were systematically executed over cycles ranging from 16 to 28 under specific conditions: initial denaturation at an elevated temperature of 98 °C sustained for 5 s; annealing maintained at 60 °C for 20 s; and extension at 72 °C for 10 s, followed by a final extension at 72 °C for 10 min. A QIAquick PCR Purification Kit from Qiagen was applied to products in the purification phase, and sequencing was then performed using the Illumina Novaseq6000 platform (Illumina, San Diego, CA, USA) (adaptors and primers sequences are listed in Table A1).

Initial raw reads were subjected to a filtration process to isolate specific digested fragments, or enzyme reads, identifiable by the activity of the BcgI restriction enzyme. Clean reads were derived by eliminating reads with greater than 8% unknown bases and discarding low-quality reads containing more than 20% low-quality bases with a quality score below Q30. In the bioinformatics pipeline (built-in Perl scripts: https://github.com/shihuang047/2bRAD-M), the species-resolved compositional profile for each sample was acquired by utilizing the 2bRAD-M sequencing data, which comprises 32 bp long reads [22]. This pipeline is dependent on a specialized 2bRAD tag database that includes taxa-specific tags derived from an extensive collection of 258,406 microbial genomes organized into 47,894 species clusters, from the Genome Taxonomy Database (GTDB R202), which encompasses bacteria, archaea, and fungi (https://gtdb.ecogenomic.org/stats/r202) [24]. A prebuilt 2bRAD species-specific marker database was employed to recognize each microbial species. The abundance of each species was calculated in an approximation based on the sequencing coverage of its species-specific markers.

Alpha diversity indices, including the Simpson, Chao 1, and Shannon indices, were evaluated by taxonomic abundance profiles. These calculations were carried out using the R programming language, particularly leveraging the functionalities provided by the “vegan” package (version 2.6.4). In beta diversity analysis, estimation was made based on Jaccard, Bray–Curtis, and Euclidean distance matrices. Principal coordinate analysis (PCoA) was used to visualize the results. These calculations were carried out using the “vegan” package in R software (version 4.2.1). Pairwise comparisons using the Wilcoxon rank-sum exact test and permutational multivariate analysis of variance (PERMANOVA test) were carried out to determine the disparities in alpha and beta diversity between the four groups. The Phylogenetic Investigation of Communities by Reconstruction of Unobserved States 2 (PICRUSt2; version 2.3.0b0) method was employed to predict functional abundance based on marker gene sequences. Default settings were used in the present study to predict the abundance of KEGG (Kyoto Encyclopedia of Genes and Genomes) orthology (KO) in the sequenced samples. The microbiome functions were predicted by the KEGG analysis (https://www.genome.jp/kegg/). In order to compare the differences in KEGG function prediction between the groups, the Kruskal–Wallis test was adopted. The significance level was established at 0.05. Abundance profiles at the species level were derived to determine the MIP for each saliva microbiota. The cumulative relative abundance of all opportunistic pathogenic bacteria in a microbial community, in accordance with 300 published categories of opportunistic pathogenic bacteria by the Chinese Center for Disease Control and Prevention [23], was used to calculate the MIP, which ranged from 0 to 1. Comparison between groups was performed using analysis of variance (ANOVA) for MIP and the Kruskal–Wallis test for fungi–bacteria ratio using IBM SPSS Statistics 27.0; the significance level was determined at 0.05.

## 3. Results

In total, 24 saliva samples were collected from 12 men and 12 women in this study. The mean age of participants was 69.46 ± 6.41. After sequencing, 254,544,343 raw reads underwent filtering, resulting in 238,215,045 enzyme reads and 228,927,179 clean reads, with an average of 9,538,632 reads per sample. Ultimately, 28 phyla, 40 classes, 93 orders, 178 families, 463 genera, and 1621 species were identified.

### 3.1. Relative Bacterial Density and Composition in Denture-Wearing and Non-Denture-Wearing Individuals in Healthy and Diseased Periodontal Conditions

**Figure 1:**
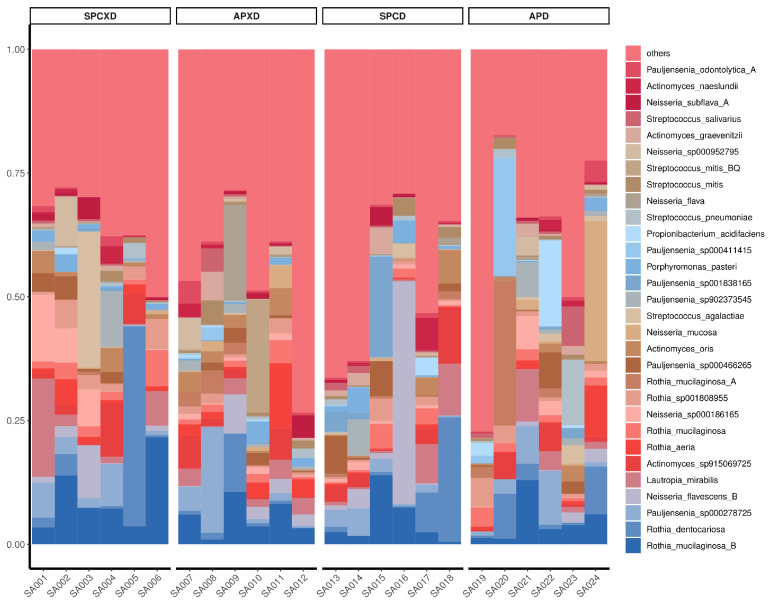
Distribution of the top 30 most abundant species in saliva samples, grouped into four categories. The predominant taxa are listed on the right side.

The top 30 species for all four groups are illustrated in Figure 1. Notably, eight pathogenic bacteria were identified, including *Streptococcus agalactiae*, *Streptococcus pneumoniae*, *Streptococcus mitis*, *Streptococcus salivarius*, *Actinomyces naeslundii*, and *Rothia* spp. Among these, *Actinomyces naeslundii* and *Rothia* spp. were identified as periodontopathic species, while *Streptococcus agalactiae* and *Streptococcus pneumoniae* were recognized as common respiratory pathogens. 

**Figure 2:**
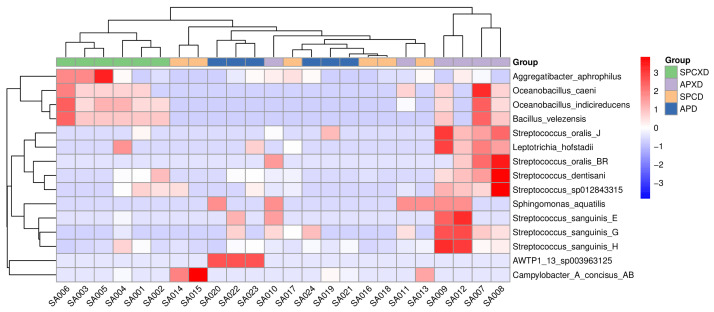
The heatmap highlights the species with significant differences in abundance between groups. Columns correspond to individual samples, while rows represent the predominant taxa. Sample groupings are displayed along the horizontal axis. Color intensity indicates the relative abundance of each taxon within the samples.

The heatmap demonstrated a distinct clustering of bacterial species within the SPCXD and APXD sample groups after taking the species with significant differences (ANOVA) in abundance between groups into consideration (Figure 2). In the SPCXD group, there was a notable prevalence of *Aggregatibacter aphrophilus*, *Oceanobacillus caeni*, *Oceanobacillus indicireducens*, and *Bacillus velezensis*. Furthermore, the heatmap revealed that the APXD samples were characterized by the common and abundant presence of *Streptococcus oralis*, *Leptotrichia hofstadii, Streptococcus dentisani*, *Sphingomonas aquatilis*, and *Streptococcus sanguinis*. Among these, *Streptococcus oralis*, *Streptococcus dentisani*, and *Streptococcus sanguinis* were recognized as pathogens. The observed clustering in the heatmap for the APXD group aligned with a microbial profile potentially associated with periodontal disease pathology. In contrast, no distinct clustering was observed in the SPCD or APD groups.

**Figure 3:**
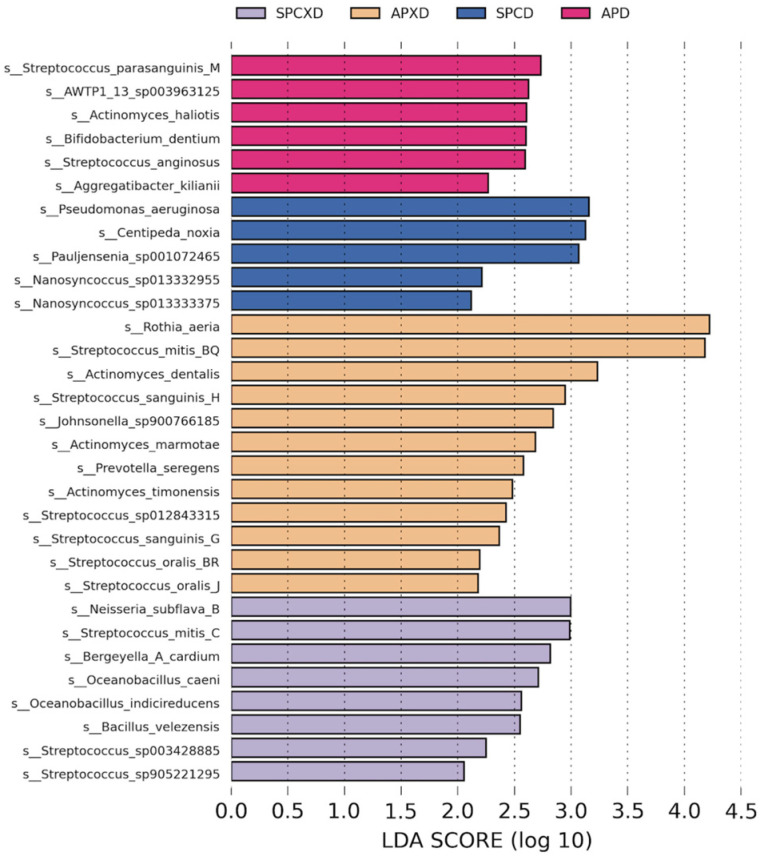
LEfSe analysis highlighting the differences in taxa abundance among the four groups. Significantly different taxa are represented by distinct colors for each group. A logarithmic LDA score threshold of 2 was set for discriminative features.

Microbial communities at the species level were compared between the four groups using LEfSe analysis (Figure 3). There were 6, 5, 12, and 8 taxa that exhibited relatively higher enrichment in the APD, SPCD, APXD, and SPCXD groups, respectively [LDA score (log10) > 2]. In the APD group, pathogenic bacteria such as *Streptococcus parasanguinis* and *Streptococcus anginosus* were identified in significant abundance. In the SPCD group, there was a significant abundance of *Pseudomonas aeruginosa*, a type of respiratory pathogen.

### 3.2. Diversity of Microbiome in Denture-Wearing and Non-Denture-Wearing Individuals in Healthy and Diseased Periodontal Conditions

**Figure 4:**
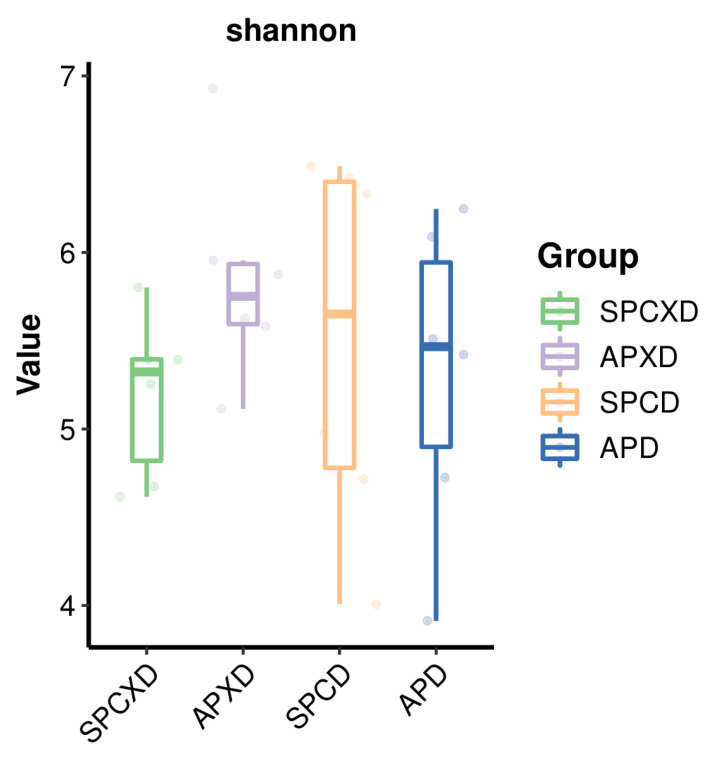
Alpha diversity analysis (Shannon index) revealed no significant difference in microbial richness between the four groups (*p* > 0.05). Each data point represents a sample, and higher values correspond to greater species diversity. Sample groups are distinguished by different colors.

**Figure 5:**
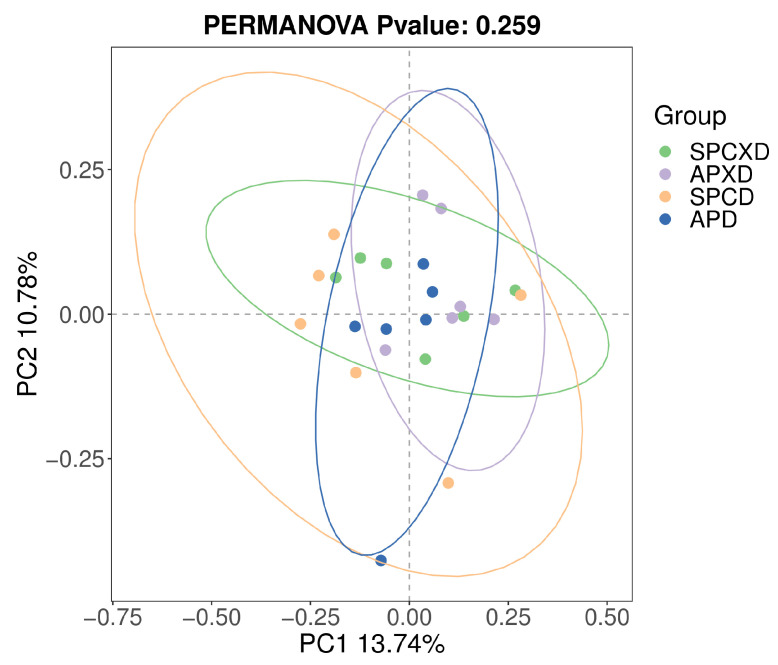
Beta diversity analysis, principal coordinate analysis based on the Jaccard (PERMANOVA; *R*^2^ = 0.14, *p* = 0.259) distance matrix. Each sample is represented by a dot. Circles in different colors represent different groups. PC1 explained 13.74% of the variation observed, and PC2 explained 10.78% of the variation.

The overall microbial richness and evenness (alpha diversity) demonstrated no statistically significant difference, as evaluated by Chao 1, Shannon, and Simpson indices at the species level (*p* > 0.05) (Figure 4). In addition, beta analyses based on Jaccard qualitative (Figure 5), Bray–Curtis quantitative distance, and Euclidean distance matrices revealed no significant differences in microbial community structures between the four groups, which was confirmed by the PERMANOVA test (Jaccard: R^2^ = 0.14, *p* = 0.259; Bray–Curtis: R^2^ = 0.11, *p* = 0.964; Euclidean: R^2^ = 0.11, *p* = 0.969).

### 3.3. Predictive Function Analysis

**Figure 6:**
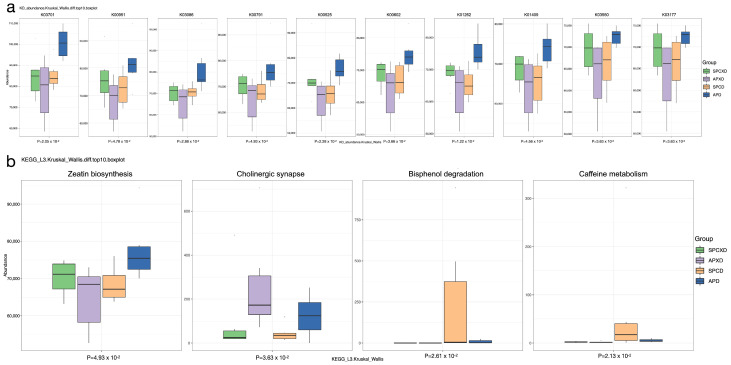
Differences in microbial predictive functions between four groups: (**a**) Differences in the predictive abundance of KO genes between groups. K03701, excinuclease ABC subunit A; K00951, GTP pyrophosphokinase; K03086, RNA polymerase primary sigma factor; K00791, tRNA dimethylallyltransferase; K00525, ribonucleoside diphosphate reductase alpha chain; K00602, phosphoribosylaminoimidazolecarboxamide formyltransferase/IMP cyclohydrolase; K01262, Xaa-Pro aminopeptidase; K01409, N6-L-threonylcarbamoyladenine synthase; K03550, Holliday junction DNA helicase RuvA; K03177, tRNA pseudouridine55 synthase. (**b**) Differences in the predictive abundance of KEGG pathways between groups.

PICRUSt2 was used to predict functional abundance based on based on sequencing data. The present study showed a noticeable increase in the predicted abundance of KO genes involved in excinuclease ABC subunit A, GTP pyrophosphokinase, RNA polymerase primary sigma factor, tRNA dimethylallyltransferase, ribonucleoside diphosphate reductase alpha chain, phosphoribosylaminoimidazolecarboxamide formyltransferase/IMP cyclohydrolase, Xaa-Pro aminopeptidase, N6-L-threonylcarbamoyladenine synthase, Holliday junction DNA helicase RuvA, and tRNA pseudouridine55 synthase in the APD group (Figure 6a). In addition, the predicted KEGG pathway that was significantly enriched in the periodontal disease groups was cholinergic synapse (Figure 6b). 

### 3.4. Fungi–Bacteria Ratio and Microbial Index of Pathogenic Bacteria

The fungi–bacteria ratio was compared using the Kruskal–Wallis test, revealing a significant difference (*p* = 0.0499) between the SPCXD and APXD groups. Moreover, a significant difference (*p* = 0.03) was detected between the SPCXD and APD groups. However, no significant difference (*p* = 0.181) was found between the SPCXD and SPCD groups. 

The mean MIP in the active periodontitis group (0.18 ± 0.13) was higher than that in the stable periodontal condition group (0.15 ± 0.09). Despite this difference, it was not statistically significant. The MIP scores for the APD, APXD, SPCD, and SPCXD groups were 0.18 ± 0.16, 0.18 ± 0.11, 0.16 ± 0.07, and 0.15 ± 0.12, respectively.

## 4. Discussion

Unclean dentures and periodontal diseases have previously been reported to be associated with various systemic diseases [6,30]. Therefore, this clinical pilot study aimed to analyze and compare the characteristics of the oral microbiome in denture-wearing and non-denture-wearing individuals with healthy and diseased periodontal conditions using high-throughput whole-metagenomic 2bRAD-M sequencing. The Human Oral Microbiome Database (HOMD) encompasses 774 identified species-level taxa in the oral cavity [31]. However, the present study identified 1621 microbial species (bacteria, archaea, and fungi). In this study, the GTDB R202 database, which comprises 258,406 genomes organized into 47,894 species clusters, was adopted to characterize the metagenomic data [24]. Additionally, the GTDB uses a new and universal taxonomy system according to genome-wide distance, while the HOMD focuses on 16S rRNA genes in the oral microbiota using the conventional NCBI taxonomy. The findings are consistent with those of Lim et al. [17] and He et al. [32]. Both studies utilized the same 2b-RAD sequencing technique for denture plaque and saliva samples, identifying more than 1900 microbial species. Notably, the 2bRAD-M was benchmarked with 16S rRNA sequencing and shotgun metagenomics methods in a previous study [22]. Therefore, the accuracy of taxonomic profiling has been well demonstrated using in silico simulation data, DNA mock community, and real clinical samples. This method also cost-effectively produced accurate, species-resolution, landscape-like taxonomic profiles for samples with high host contamination, low biomass, and degradation [17,22].

**Figure A1:**
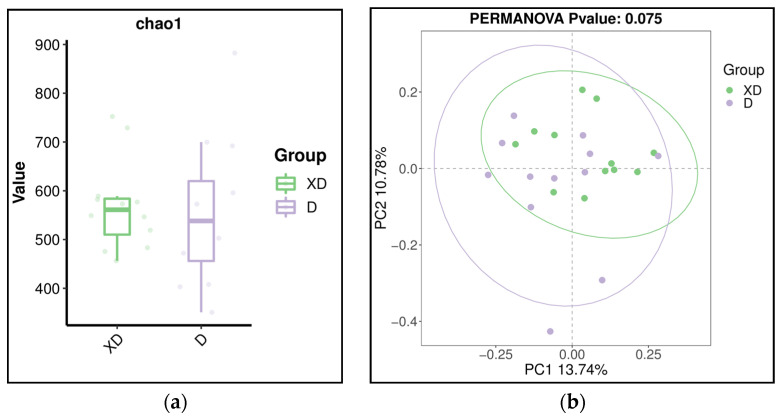
(**a**) Alpha diversity analysis (Chao 1) revealed no significant difference in microbial richness between denture-wearing and non-denture-wearing (*p* = 0.51). (**b**) Beta diversity analysis and principal coordinate analysis based on the Jaccard (PERMANOVA; R^2^ = 0.06, *p* = 0.075) distance matrix.

The present study demonstrated that APXD had greater microbial richness and evenness than SPCXD, revealing a nearly significant result (*p* = 0.065), which is consistent with studies showing that the active periodontal disease had greater index scores in alpha diversity than the healthy group [33,34]. The higher species diversity in the active periodontitis group may have resulted from strong dysbiosis that was caused by periodontopathic bacteria [35]. The present study showed no significant difference between the denture groups. Possibly, greater complexities of microorganisms were found in the denture group, as compared to the non-denture group [36]. Additionally, a review study also explained that denture cleanliness plays a pivotal role in determining the complexity of bacterial colonies found within these denture groups [14]. It was observed that denture hygiene directly correlates with species complexity [17]. The microbial community composition between groups showed no statistically significant differences considering all indices. However, a clear trend of dissimilar oral microbiomes between denture-wearing and non-denture-wearing groups based on the Jaccard distance was demonstrated (PERMANOVA test: R^2^ = 0.06; *p* = 0.075) (Figure A1). In this study, saliva samples were analyzed, and it was proposed that microorganisms attached to the teeth and mucosa surfaces continuously shed into the salivary fluid. Both stimulated and unstimulated saliva exhibited certain similarities with the tongue and buccal mucosa. However, there was no correlation observed between the bacterial composition found in saliva and that described in the supra- or subgingival dental plaque, which may not easily shed, particularly thick plaque that houses obligate anaerobes, which may not dislodge easily with saliva [37]. The present finding is also consistent with another study investigating the bacterial community associated with healthy denture wearers and denture wearers with denture stomatitis. The beta diversity was also not statistically significant among the groups including healthy denture wearers and non-denture wearers [16]. 

The heatmap analysis demonstrated a pronounced clustering of specific microbial species with significant differences in abundance within the negative control group (SPCXD), including *Aggregatibacter aphrophilus*, *Oceanobacillus caeni*, *Oceanobacillus indicireducens*, and *Bacillus velezensis*. None of these species were listed in the microbial index of pathogenic bacteria, indicating their non-pathogenic nature. The heatmap analysis also revealed another distinct clustering of microbial species found in the APXD group. Among these species, *Leptotrichia hofstadii* has been isolated from subgingival samples and gingival crevicular fluid in periodontitis cases. *Streptococcus oralis* has been recognized as a commensal organism in the oral cavity and an opportunistic pathogen capable of invading the bloodstream to cause bacteremia and infective endocarditis [38]. *Streptococcus dentisani* has been identified as a common inhabitant of the human oral cavity and possesses the ability to inhibit periodontal pathogens through a comprehensive probiotic action, characterized by antimicrobial, anti-adhesive, and anti-inflammatory effects [39]. Another pathogen found in the APXD group clustering is *Streptococcus sanguinis*, which is a Gram-positive, non-spore-forming, facultative anaerobic bloodstream pathogen that may cause infective endocarditis [40]. 

Among the top 30 species residing in saliva samples, many pathogenic bacteria, including *Streptococcus agalactiae*, *Streptococcus pneumoniae*, *Streptococcus mitis*, *Streptococcus salivarius*, *Actinomyces naeslundii*, and *Rothia* spp., were identified. Notably, *Streptococcus agalactiae* is known for its potential to induce a wide range of invasive conditions. These can include bacteremia, which may result in cardiac valve infections and endocarditis, as well as skin and soft tissue infections, both acute and chronic osteomyelitis, pneumonia, meningitis, and urinary tract infections [41]. *Streptococcus pneumoniae*, another keystone pathogen identified, is a Gram-positive, opportunistic, and extracellular bacterium that resides in the mucosal surfaces of the human upper respiratory tract. This bacterium may maintain a commensal relationship with the host. However, when it disseminates from its primary site along the nasal epithelium via aspiration, bacteremia, or local proliferation, it is capable of leading to severe invasive diseases such as pneumonia, meningitis, and otitis media [42]. Furthermore, the detection rate of Streptococcus pneumoniae was relatively high in denture plaque [18,21]. This indicated that Streptococcus pneumoniae could not only colonize the mucosal surfaces of the human upper respiratory tract but also establish itself within dental prostheses. In addition, several periodontopathic species were identified including *Actinomyces naeslundii* and *Rothia* spp. 

LEfSe analysis revealed the substantial presence of pathogenic bacteria in the APD group, including *Streptococcus parasanguinis* and *Streptococcus anginosus*. *Streptococcus parasanguinis* is closely associated with the development of infective endocarditis [43]. *Streptococcus anginosus*, in addition to its role as a pathogenic bacterium, has been implicated in the induction of pyogenic infections [44]. There was a significantly higher abundance of *Pseudomonas aeruginosa*, a recognized pathogen, detected in the SPCD group. This bacterium has been implicated in severe nosocomial pneumonia among hospitalized and institutionalized individuals, particularly affecting those with compromised immune systems, leading to substantial morbidity and mortality [45]. *Pseudomonas aeruginosa* was found as the predominant species in denture plaque, highlighting its abundance in comparison to other species [19,20]. These observations are in line with our findings, which show an elevated presence of *Pseudomonas aeruginosa* in the denture group. 

Denture plaque has been shown to exhibit a microbial composition similar to dental plaque but with higher levels of fungi (particularly *Candida* spp.), *Lactobacillus* spp., *Streptococci*, and *Staphylococci* [14]. These elevated levels have been specifically observed in cases of denture stomatitis and have been found to increase as the denture ages [46]. The significant increase in the fungi–bacteria ratio in active periodontitis groups suggests a possible relationship between fungi and periodontitis. This finding is consistent with a systematic review, which showed that *Candida* spp. may increase the risk of periodontal disease development by 1.76 times [47]. *Candida albicans* can interact with periodontopathic pathogens to develop mixed biofilms, suggesting that fungi may play an active role in the inflammatory destructive process during the onset and progression of periodontal diseases [48]. In addition to taxonomic analyses, this study also investigated the MIP in denture wearers with different periodontal statuses. This index measures the potential pathogenicity of the microbial community, calculated based on the abundance of known pathogenic bacterial species. As expected, the MIP was found to be higher in groups with periodontal diseases compared to those with healthy periodontal status, suggesting a potential association between MIP and periodontal diseases [26]. However, the difference was not significant. The observed differences in the predicted abundance of KO genes between groups demonstrated a marked increase in the predicted abundance of specific genes in the APD group. However, none of these genes have a direct, well-established relationship with periodontal diseases. The predicted KEGG pathway that was significantly enriched in the periodontal disease groups was cholinergic synapse, which is consistent with the finding of Zhou et al. [49]. We did not aim to perform predictive modeling of any oral and systemic infections. Therefore, prospective clinical studies with larger sample sizes evaluating human health using the MIP and predictive function analysis are recommended to overcome the limitations of existing evidence. In addition, the role of keystone taxa, which are low in abundance but ecologically significant, should not be underestimated [50]. The present study detected several keystone pathogens in saliva samples, including *Streptococcus agalactiae*, *Streptococcus pneumoniae*, and *Pseudomonas aeruginosa*. These pathogens are known to be associated with respiratory infections and play a significant role in pneumonia due to microbial dysbiosis and the disruption of host-microbial homeostasis. The microbial activity and virulence of specific keystone pathogens may contribute to an increased risk of carcinogenesis, as previously reported, which supports the relationship between microbial dysbiosis and disease development [51]. However, it is crucial to determine the significance of the observed high abundance of potential pathogens within the oral microbiome, as it raises concerns about the overall health of the host, as demonstrated in this study. 

In this pilot study, a small number of community-dwelling elders were recruited due to the study’s preliminary nature. The main limitation of this study is selection bias, which resulted from the use of convenience sampling. However, to counterbalance the sample size and selection bias limitations, relatively strict inclusion and exclusion criteria were employed. The clinical significance of this pilot study, which discovered respiratory pathogens in saliva samples of older adults, lies in its implications for both dental and medical disciplines, potentially influencing the management of care for older individuals. Furthermore, future microbiome studies should be conducted to assess changes in metagenomic characterization following denture cleaning interventions or periodontal treatments.

## 5. Conclusions

In the cohort of saliva samples collected from community-dwelling elders, a remarkable abundance of opportunistic pathogens was detected within the microbial community. This significant finding highlights the imperative for enhanced medical protection measures among older adults, particularly those wearing removable dentures and having active periodontal diseases.

## Figures and Tables

**Table 1:** Inclusion and exclusion criteria.

Inclusion Criteria (for Participants Aged 60 and above)	
(i) Diseased periodontal condition	
1.	Participants without evidence of previous therapy and clinical improvement:
	Interdental clinical attachment loss (CAL) was detectable at ≥2 non-adjacent teeth, OR
	Buccal or oral CAL ≥ 3 mm at ≥2 teeth
2.	Participants who had been given periodontal therapy and presented with one or more non-responding periodontal pocket sites:
	Periodontal pocket depth ≥ 5 mm
	Periodontal pocket depth ≥ 4 mm with bleeding on probing (BOP)
(ii) Healthy periodontal condition	
1.	Participants without sign and history of periodontitis, characterized by the absence of detectable attachment and bone loss
2.	Participants with gingival health on a reduced periodontium, characterized by intra-oral BOP < 10%, periodontal pocket depth ≤ 4 mm, and no 4 mm periodontal pocket depth with BOP
3.	Participants with gingival inflammation in a stable periodontitis patient, characterized by intra-oral BOP > 10%, periodontal pocket depth ≤ 4 mm, and no 4 mm periodontal pocket depth with BOP
(iii) Removable denture groups	
1.	Denture base made of acrylic resin
2.	Partially dentate
3.	Good denture quality with Kapur index of retention score ≥ 2 and stability score ≥ 1
4.	Denture cleanliness index ≥ 1 (denture has visible plaque and/or debris with ≥25% fit surface stained)
**Exclusion Criteria**	
1.	Rinse with mouthwash prior to sample collection
2.	Mentally, physically, or medically unfit (dementia and neurological diseases, received radiotherapy or chemotherapy, use of steroid treatment for more than 2 weeks in the past year, use of local and/or systemic antibiotic therapy/antifungal/antiviral/anti-inflammatory/bisphosphonates/phenytoin within 1 month prior to sample collection)
3.	Use of denture adhesive products or the acrylic denture was relined
4.	Currently smoking or quit smoking less than 6 months

## Data Availability

As of the date of publication, the data from this study are publicly available in the NCBI Short Read Archive (SRA) under BioProject ID number PRJNA1121417.

## Appendix A

**Table A1:** Adaptors and primers used for 2bRAD-M library preparation.

Adaptor	Sequence (5′ to 3′)
Adap-1 sense	ACACTCTTTCCCTACACGACGCTCTTCCGATCTNNN
Adap-1 antisense	AGATCGGAAGAGC(AminoC6)
Adap-2 sense	GTGACTGGAGTTCAGACGTGTGCTCTTCCGATCTNNN
Adap-2 antisense	AGATCGGAAGAGC(AminoC6)
Primer	
Primer1	ACACTCTTTCCCTACACGACGCT
Primer2	GTGACTGGAGTTCAGACGTGTGCT
Primer3	AATGATACGGCGACCACCGAGATCTACACTCTTTCCCTACACGACGCT
Index primer	CAAGCAGAAGACGGCATACGAGATXXXXXXGTGACTGGAGTTCAGACGTGT
